# Buruli ulcer disease prevalence in Benin, West Africa: associations with land use/cover and the identification of disease clusters

**DOI:** 10.1186/1476-072X-7-25

**Published:** 2008-05-27

**Authors:** Tyler Wagner, M Eric Benbow, Travis O Brenden, Jiaguo Qi, R Christian Johnson

**Affiliations:** 1 Quantitative Fisheries Center, Department of Fisheries and Wildlife, Michigan State University, East Lansing, MI 48824, USA; 2 U.S. Geological Survey, Pennsylvania Cooperative Fish & Wildlife Research Unit, Pennsylvania State University, 402 Forest Resources Bldg, University Park, PA 16802, USA; 3 Department of Entomology, Michigan State University, East Lansing, MI 48824, USA; 4 Center for Global Change and Earth Observations, Michigan State University, East Lansing, MI 48824, USA; 5 Programme National de lutte contre l'UB, Cotonou, Benin

## Abstract

**Background:**

Buruli ulcer (BU) disease, caused by infection with the environmental mycobacterium *M. ulcerans*, is an emerging infectious disease in many tropical and sub-tropical countries. Although vectors and modes of transmission remain unknown, it is hypothesized that the transmission of BU disease is associated with human activities in or around aquatic environments, and that characteristics of the landscape (e.g., land use/cover) play a role in mediating BU disease. Several studies performed at relatively small spatial scales (e.g., within a single village or region of a country) support these hypotheses; however, if BU disease is associated with land use/cover characteristics, either through spatial constraints on vector-host dynamics or by mediating human activities, then large-scale (i.e., country-wide) associations should also emerge. The objectives of this study were to (1) investigate associations between BU disease prevalence in villages in Benin, West Africa and surrounding land use/cover patterns and other map-based characteristics, and (2) identify areas with greater and lower than expected prevalence rates (i.e., disease clusters) to assist with the development of prevention and control programs.

**Results:**

Our landscape-based models identified low elevation, rural villages surrounded by forest land cover, and located in drainage basins with variable wetness patterns as being associated with higher BU disease prevalence rates. We also identified five spatial disease clusters. Three of the five clusters contained villages with greater than expected prevalence rates and two clusters contained villages with lower than expected prevalence rates. Those villages with greater than expected BU disease prevalence rates spanned a fairly narrow region of south-central Benin.

**Conclusion:**

Our analyses suggest that interactions between natural land cover and human alterations to the landscape likely play a role in the dynamics of BU disease. For example, urbanization, potentially by providing access to protected water sources, may reduce the likelihood of becoming infected with BU disease. Villages located at low elevations may have higher BU disease prevalence rates due to their close spatial proximity to high risk environments. In addition, forest land cover and drainage basins with variable wetness patterns may be important for providing suitable growth conditions for *M*. *ulcerans*, influencing the distribution and abundance of vectors, or mediating vector-human interactions. The identification of disease clusters in this study provides direction for future research aimed at better understanding these and other environmental and social determinants involved in BU disease outbreaks.

## Background

Emerging and re-emerging infectious diseases are an increasing health concern for many parts of the world. Between 1972 and 1999, 35 new disease-causing agents were identified, with many more diseases re-emerging after years of latency [1]. Understanding the ecology of these pathogens and the environmental and social factors that drive disease dynamics is difficult because of the complex nature of the factors involved in disease processes, including changes in human demography, human behaviour, global climate, and anthropogenic alterations to the landscape [2]. The lack of a mechanistic understanding of how environmental and social conditions interact with disease processes to ultimately cause human infections can severely hinder prevention and control programs.

Further limiting our ability to prevent disease spread is the fact that many of the factors influencing emerging diseases are difficult and expensive to quantify. Case-control studies, whereby the risk factors for those infected with a disease are compared to those who have not been affected, provide invaluable information on modes of disease transmission. However, these studies are often conducted at limited spatial scales, which can prevent the identification of large-scale spatial patterns in disease prevalence. Conversely, spatial epidemiological studies of disease, whereby the geographic distribution of diseases are described and analyzed [3], can be a useful and cost-effective first step for preventing disease spread for several reasons. First, infectious diseases are often constrained spatially by local and regional environmental factors, and analyses of large-scale patterns in disease prevalence can help identify factors that may influence disease spread. For example, landscape features such as land use/cover types can directly or indirectly influence a pathogen's ability to survive in the environment or be transmitted by influencing reservoir and vector dynamics (e.g., by influencing abiotic conditions and biotic interactions) and/or the probability of encountering a human host (e.g., by influencing human behaviour). Additionally, the land use/cover data (and other map-based data) are now widely available due to advances in Geographic Information Systems (GIS) and remote sensing technology. These technologies offer the ability to collect vast amounts of data over large spatial regions when other, often finer resolution, data are unavailable or in the process of being collected. Spatial epidemiological studies have proven useful for understanding the geographical distribution and landscape-drivers of many diseases, including Puumala virus, Lyme borreliosis disease, malaria, and Human African Trypanosomosis, among others [4-8].

An emerging infectious disease for which there exists a great amount of uncertainty and for which spatial epidemiological studies may yield important clues regarding disease spread is Buruli ulcer (BU) disease [9,10]. Buruli ulcer disease is caused by the environmental mycobacterium *M. ulcerans*, and is the third most common mycobacterial infection of humans after tuberculosis and leprosy (although Renzaho et al. suggest that BU disease is now the second most prevalent mycobacterial disease in Ghana, Africa [11]). Buruli ulcer disease has been reported from at least 31 tropical and sub-tropical countries, with the West Africa countries of Côte d'Ivoire, Ghana, and Benin, being particularly affected. Buruli ulcer disease manifests itself as chronic ulcerations or plaques of the skin, with the bone in some cases also being affected [12,13]. Severe cases can result in contracture deformities and amputation, leading to substantial socioeconomic hardship for those inflicted [14]. Case-control studies have identified the use of unprotected water from swamps [15,16] and rivers [17,18], particularly systems that have been heavily impacted by agricultural land use within surrounding catchments [19,12], as risk factors for BU disease. In addition, an impaired immune system, potentially as a result of exposure to soil or water enriched with naturally occurring elements or anthropogenic contaminants, is also a risk factor for BU disease [20]. For example, Duker et al. [21-23] demonstrated spatial relationships between BU prevalence and arsenic (an immunosuppressant agent) in the Amansie West district, Ghana. Specifically, Duker et al. [21] found that mean BU prevalence was higher in arsenic-enriched drainages and farmlands compared to areas elsewhere in the district. A positive exposure-response relationship between arsenic in surface water and BU prevalence has also been demonstrated [22]. Although these (and other) risk factors have been identified, the exact mechanism by which humans become infected with *M. ulcerans *in or near aquatic habitats remains unknown. One hypothesis is that *M. ulcerans *is transmitted through skin abrasions or other skin injuries after contact with water, vegetation, or soil. In addition, anecdotal evidence suggests that biting aquatic insects (Hemiptera) may be involved in the transmission of BU disease [24], but this has yet to be confirmed in humans, and recent field data suggest that this hypothesis is unlikely [25].

The purpose of this research was to attempt to better understand the geographical distribution of BU disease in Benin, West Africa, which has been severely affected by this disease (more than 4000 cases reported between 1989 and 2000; [26]). Specific objectives of our research were (1) to determine whether BU disease prevalence was related to surrounding land use/cover of Benin villages and (2) to identify disease clusters and land use/cover types associated with BU disease prevalence within disease clusters to assist in the development of prevention and control programs. To our knowledge, ours is the first study that has examined BU disease prevalence across a large spatial area, including most of the country of Benin. The results of this study should provide invaluable information pertaining to the spatial epidemiology of BU disease.

## Methods

### Study area

**Figure 1:**
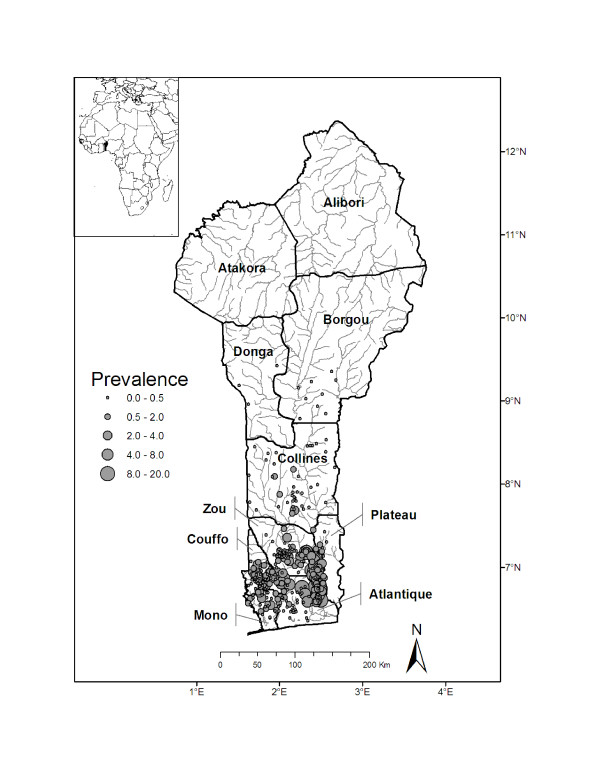
**Study map**. Study map of Benin showing districts, individual villages, and Buruli ulcer disease prevalence rates (per 1,000 individuals).

The Republic of Benin encompasses 112,622 km^2 ^and has an estimated human population of nearly eight million people. Benin is bordered by Burkina Faso and the Republic of Niger to the north, by the Federal Republic of Nigeria to the east, and by the Republic of Togo to the west. To the south of Benin lies the Gulf of Guinea (Figure 1).

### Buruli ulcer disease case data

Buruli ulcer disease prevalence from 327 villages in Benin in 2004 and 2005 were obtained from BU 02 surveillance forms. These forms, which provide community-based surveillance data of BU patients [27], were developed by the World Health organization as a means for obtaining standardized reporting of diseases within and across counties (WHO 2000).

### Map-based covariates

Land use/cover in regions surrounding Benin villages were summarized using Landsat ETM+ imagery obtained from the University of Maryland Global Land Cover Facility [28], December 13, 2000. After geometrically rectifying and projecting the imagery, an unsupervised classification of the imagery bands was performed in Erdas IMAGINE with 100 initial classes on the principal axis with pseudo-color for ten iterations or a 0.95 convergence [29]. This resulted in 22 land use/cover categories, which were subsequently narrowed to five unique land use/cover categories. These five land use/cover categories included percent agriculture, urban, water, forest, and wetland land use/cover types in regions surrounding each Benin village. The percentages of these land use/cover categories were summarized using circular buffers created around each Benin village in ArcGIS [30]. Radii of 0.1, 0.5, 1, 5, 10, 20, and 50 km were used to create these buffers so that the effect of various spatial scales of land use on BU disease prevalence could be examined. The percent of each land use/cover category was calculated by dividing the number of pixels for each individual land use/cover class within a buffer by the total number of pixels within that buffer.

In addition to the percent of land use/cover categories in surrounding regions, we also calculated the compound topographic index, otherwise known as the wetness index [31,32], as a measure of landscape wetness for Benin villages. The wetness index was calculated using a flow accumulation (FA) layer along with the slope as:

(1) Wetness index = ln(*FA*/tan(*slope*))

In areas of no slope, a wetness index value is obtained by substituting a slope of 0.001. This value is smaller than the smallest slope obtainable from a 1000 m data set with a 1 m vertical resolution. Both the mean wetness index and the standard deviation of the wetness index for the drainage basin for each village were calculated. We also included a measure of village mean elevation above sea level as an indicator of whether or not a village was located in lower elevation areas where standing water might accumulate. The distance of each village to its nearest river was calculated using a river coverage at a 1:1,000,000 scale. The distance to the nearest river was calculated using all the water feature types included in this coverage; thus, measurements included distances to streams, rivers, and channelized rivers.

Based on case-control studies and our current understanding of BU disease, we made the following predictions regarding how our measured landscape features would affect BU disease prevalence in villages in Benin. We predicted that percent agriculture land use, water, and wetland cover surrounding villages would be associated with high prevalence due to the increased risk associated with exposure to aquatic habitats and agricultural activities near water. We also predicted that percent urban land use and forest land cover would be associated with lower prevalence, as urban environments provide greater access to pumped water sources, and forest land cover potentially indicates less human disturbance to the landscape. Lastly, we predicted that villages located in basins with variable wetness patterns at low elevations, and near rivers would have higher prevalence rates. We hypothesized that variable wetness patterns would establish favourable conditions for the establishment and growth of *M. ulcerans*, and that villages at low elevations and near rivers would have higher prevalence due to close spatial proximity to high risk environments.

### Statistical modelling

Using the explanatory variables described above (Table 1), we developed a set of models that attempted to relate BU disease prevalence rates to village landscape features. BU disease prevalence rates were related to village landscape features using negative binomial regression models. Like Poisson regression models, negative binomial regression models are intended for modelling count data. However, unlike the Poisson distribution which is characterized by a single parameter (*μ*), which is both the mean and variance (*E*(*Y*) = *Var*(*Y*) = *μ*) for the distribution, the negative binomial has an additional parameter (*k*) which allows the variance to exceed the mean (*E*(*Y*) = *μ*, *Var*(*Y*) = *μ *+ *kμ*^2^. Thus, negative binomial regression models are preferred over Poisson models when the variance of the count data is larger than the mean. The number of BU disease cases in an individual village was the response variable, and the natural logarithm of the total population size of each village was included as an offset term in the model. To account for possible correlation of BU prevalence for villages within the same Benin district, we included a random district effect within our model [33,34]. Although we did not hypothesize that BU prevalence rates were constrained by district boundaries, we did hypothesize that districts would differ in their average BU prevalence based on district differences in environmental factors such as rainfall and social factors such as the amount of public health education. The general form of the model is as follows:

**Table 1:** Summary statistics for the number of Buruli ulcer cases and potential covariates used in analyses.

Variable	Mean	Minimum	Maximum
Dependent variable			
Buruli ulcer cases	1.8	0.0	29
Covariates			
Agriculture (0.1)	0.33	0.0	1.0
Agriculture (0.5)	0.33	0.0	1.0
Agriculture (1)	0.33	0.0	1.0
Agriculture (5)	0.33	0.0	0.88
Agriculture (10)	0.33	0.0	0.77
Agriculture (20)	0.31	0.0	0.61
Agriculture (50)	0.25	0.0	0.42
Urban (0.1)	0.05	0.0	1.0
Urban (0.5)	0.03	0.0	0.62
Urban (1)	0.02	0.0	0.48
Urban (5)	0.01	0.0	0.21
Urban (10)	0.01	0.0	0.13
Urban (20)	0.01	0.0	0.06
Urban (50)	0.01	0.0	0.06
Water (0.1)	0.0	0.0	1.0
Water (0.5)	0.01	0.0	0.88
Water (1)	0.01	0.0	0.72
Water (5)	0.01	0.0	0.42
Water (10)	0.01	0.0	0.34
Water (20)	0.01	0.0	0.23
Water (50)	0.01	0.0	0.07
Wetland (0.1)	0.0	0.0	0.17
Wetland (0.5)	0.01	0.0	0.19
Wetland (1)	0.01	0.0	0.20
Wetland (5)	0.0	0.0	0.18
Wetland (10)	0.02	0.0	0.23
Wetland (20)	0.02	0.0	0.17
Wetland (50)	0.03	0.0	0.11
Forest (0.1)	0.02	0.0	1.0
Forest (0.5)	0.03	0.0	0.71
Forest (1)	0.03	0.0	0.50
Forest (5)	0.04	0.0	0.57
Forest (10)	0.05	0.0	0.70
Forest (20)	0.06	0.0	0.76
Forest (50)	0.07	0.0	0.52
Distance to river (m)	22.4	3,850	12,157
Wetness index	6.8	7.9	9.2
Mean elevation (m)	120.9	51.8	345.4

Land use/cover was estimated for different buffer widths around individual villages, while mean wetness index and elevation are based on the village drainage basin (see Methods). Land use/cover variable is followed by buffer radius (km) in parentheses.

(2) $$\log(Y_{ij})=\log(N_{ij})+\beta_{0}+a_{j}+\sum_{q=1}^{Q}\beta_{q}X_{qij}$$

where log(*Y*_*ij*_) is the expected outcome for the dependent variable *y *for village *i *in district *j*, log(*N*_*ij*_) is the natural logarithm of the total population for village *i *in district *j *(the offset term), the parameter *β*_0 _is the intercept, and *β*_*q *_is the effect of covariate *X*_*qij *_on the dependent variable, where *Q *is the total number of covariates. The district-specific random effect is defined as *a*_*j *_~ *N*(0,$\sigma_{d}^{2}$), where $\sigma_{d}^{2}$ is represents the variance between districts. When fitting models, covariates measured as proportions were arcsine-square root transformed and elevation and the wetness index were natural log transformed.

Briefly our model building process was as follows: (1) although we had hypotheses for the effects of the covariates on BU disease, we needed an approach to reduce the potential set of covariates prior to constructing a set of candidate models. To accomplish this, each covariate was modelled separately and assessed by comparing the value of the Akaike's Information Criterion (AIC) [35] of the model with a single covariate to a model that included only a random intercept and the offset term. AIC is an information-theoretic approach to assessing model fit that accounts for both bias and precision. The model with the lowest AIC value is the best fitting model of the candidate models being considered. We used the AIC adjusted for small sample size (AICc) [36] because the ratio of the number of observations to the number of parameters estimated in the highest-dimensioned model was near 40 [36]; (2) those covariates that resulted in a lower AICc value relative to the random intercept model were retained for consideration in the construction of multivariate models; (3) because the land use/cover of the same type in each buffer width (e.g., percent agriculture measured at each of the 7 buffer widths) were highly correlated, the land use/cover variable at the buffer width (e.g., percent agriculture within a 20 km buffer) that resulted in the largest reduction in AICc was retained for the construction of multivariate models; (4) after reducing our set of potential covariates, they were then assessed for multicollinearity; (5) lastly, using the non-correlated covariates we constructed a set of candidate models (i.e., hypotheses) [see Additional file 1]. We compared the set of candidate models by calculating AICc weights and strength of evidence for each model [36]. AICc weights (*w*_*i*_) for model *i *were calculated as

(3) $$w_{i}=\frac{e^{\left(-\frac{1}{2}\Delta_{i}\right)}}{\sum_{r=1}^{R}e^{\left(-\frac{1}{2}\Delta_{r}\right)}}.$$

where Δ_*i *_is the difference between the AICc for the i^th ^model and the minimum AICc for all models. The AICc weights are a measure of each model's relative probability of being the best model, given the R models considered [36]. The strength of evidence was calculated as *w*_max_/*w*_*i *_for each model, where *w*_max _is the largest weight for all candidate models and *w*_*i *_is the weight for the model *i*. Model estimation was performed using the glmmADMB package in the R software [37].

To determine whether there was any remaining spatial variation in BU prevalence data, we examined the empirical semivariogram of the residuals (residuals = [observed – fitted]/sd, where $\text{sd}=\sqrt{fitted\times (1+fitted)/\alpha }$ and *α *is the estimated negative binomial parameter) from our best-performing negative binomial regression model. The presence of remaining spatial variation would indicate whether we had omitted an important spatially-varying covariate from our model or to the possibility of spatial autocorrelation among our observed data. To test the statistical significance of any remaining spatial variation in our data, we used simulations (*n = *99) to construct empirical semivariogram envelopes. These simulations were performed under the null hypothesis of spatial independence; thus, any observation could be assigned to any of the observed village locations. The semivariogram envelopes are constructed by taking the minimum and maximum semivariances at each lag distance. If all points from the empirical semivariogram fall within the simulation envelopes, then the null hypothesis of spatial independence in not rejected [38]. The empirical semivariogram and the semivariogram envelopes were estimated using the geoR package [39] in R.

### Disease cluster detection

We used the spatial scan statistic [40] as implemented in SaTScan [41] to detect and test the statistical significance of spatial clusters of high and low rates of BU disease prevalence in Benin villages. A Poisson probability model and circular spatial window was used for the spatial scan analysis. The maximum radius of the circular spatial windows was such that the window could not include more than 50% of the total population at risk. In addition to the most likely cluster, secondary clusters also were identified as part of the spatial scan analysis. For the secondary clusters, only clusters that did not geographically overlap other identified clusters were reported. We decided not to allow secondary clusters to overlap (i.e., any one village could only be associated with a high or low cluster) in order to ease interpretation. Tests of statistical significance of the identified clusters were based on likelihood ratio tests, with *P-*values obtained by Monte Carlo randomization [42].

To investigate if different landscape covariates were related to BU disease prevalence in clusters with greater or lower than expected prevalence rates, we used a similar modelling approach to the one described above (see Statistical Modelling). The model was similar in structure to equation 1; however, we excluded the random district effect because clusters did not encompass multiple districts. AICc weights for competing models were also calculated, and we examined the residuals for autocorrelation as described above.

## Results

### Association of Buruli ulcer disease with land use/cover types

**Figure 2:**
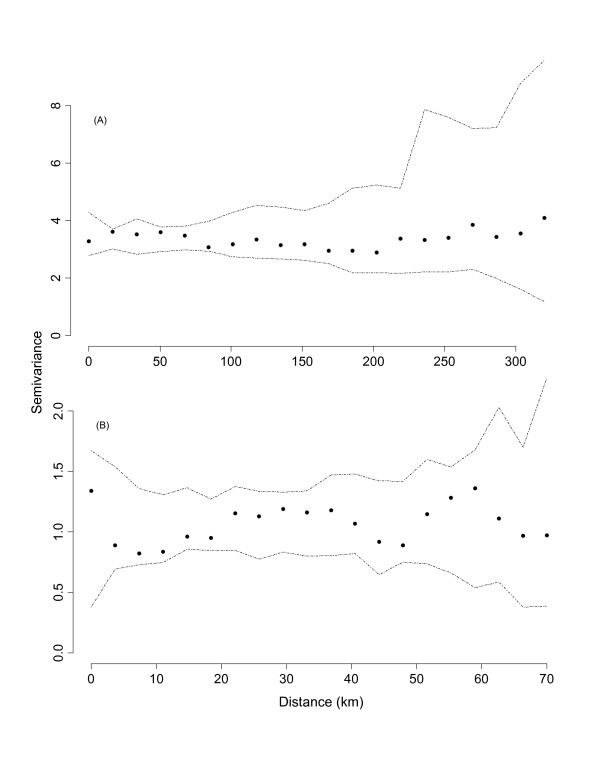
**Empirical variogram and envelopes of residuals from the top-ranked (A) and disease cluster (B) negative binomial regression models**. Residuals are from a negative binomial regression model relating Buruli ulcer disease prevalence to landscape attributes in Benin. Envelopes were computed by permutation of the residual values across spatial locations.

The number of BU disease cases in villages ranged from 0 to 29 (mean = 1.7, variance = 9.1), with the majority of cases occurring in the southern portion of Benin (Figure 1). The following covariates were significantly correlated and thus not included in the same model: percent agriculture land use and forest cover in a 20 km buffer (r = -0.62) and mean elevation and wetness index (r = -0.88). Of the models that we constructed, AICc ranged in value from 1114.6 to 1145.4. The model with the lowest AICc contained 4 covariates: percent urban land use (20 km buffer), percent forest land type (20 km buffer), mean elevation of village drainage basin, and the standard deviation of the wetness index. Based on the AICc strength of evidence, this model was 2.3 times more likely than the next best-performing model [see Additional file 1]. As expected, percent urban land use and mean elevation were negatively correlated, and the standard deviation of the wetness index was positively correlated with BU disease prevalence (Table 2). Contrary to our predictions, percent forest cover was positively correlated with BU disease prevalence. Furthermore, examination of the empirical variogram envelopes of the residuals from the top-ranked model suggested that the null hypothesis of spatial independence was not rejected (Figure 2A). Thus, our non-spatial model appeared adequate and a more complex model was not deemed necessary.

**Table 2:** Final model parameter estimates for negative binomial regression models.

Model	Parameter estimates					
Country-wide analysis	Intercept	Urban (20)	Forest (20)	Mean elevation	Wetness index standard deviation	Random district effect ($\sigma_{d}^{2}$)
	0.76 (1.71)	-3.62 (1.96)	1.68 (0.88)	-0.85 (0.22)	3.06 (1.27)	0.55 (0.37)
Cluster 1	Intercept	Urban (0.5)				
	-6.05 (0.14)	-5.28 (1.81)				

**Figure 3:**
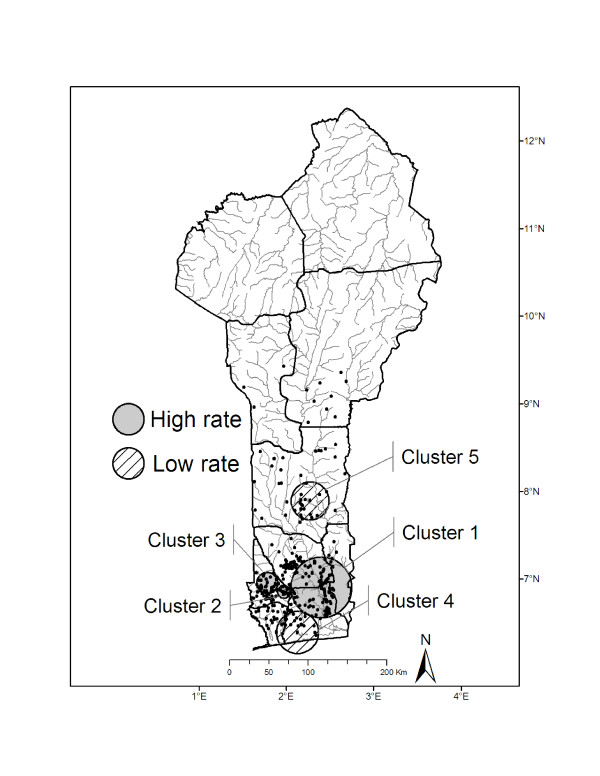
**Results of spatial cluster analysis**. Spatial cluster analysis identified one primary cluster with greater than expected Buruli ulcer disease prevalence rates (cluster 1) and four secondary clusters: two with greater than expected prevalence rates (clusters 2 and 3) and two with lower than expected prevalence rates (clusters 4 and 5).

Response variable is the number of Buruli ulcer cases in villages in Benin, West Africa. Models for two analyses are presented: a country-wide analysis (*n *= 327 villages), and an analysis for Buruli ulcer disease in cluster 1 (see methods;* n *= 100 villages). Cluster 1 had greater than expected prevalence (see Figure 3 for location of cluster). Parameter estimates are followed by standard errors in parentheses. Land use/cover types are percent in a buffer surrounding each village, the buffer width (km) follows the land use/cover type in parentheses.

### Buruli ulcer disease clusters

The spatial scan analysis identified five significant BU disease clusters (*P *< 0.0001 for all clusters) for the Benin villages (Figure 3). The primary cluster had greater than expected prevalence rates and included 100 villages along the Zou and Ouémé rivers. The two secondary clusters with greater than expected prevalence were located to the west of cluster 1 and included 15 villages in cluster 2 and 31 villages in cluster 3. These clusters included villages along the Couffo River (Figure 3). The two clusters that were identified as having lower than expected prevalence rates were located near the coast (cluster 4, n = 23 villages) and to the north of the other clusters in the Collines district (cluster 5, n = 18 villages).

Because of small sample sizes (number of villages) in secondary clusters, we were only able to fit models for the primary cluster, cluster 1. Negative binomial regressions identified a single covariate correlated with BU disease prevalence for the primary cluster (Table 2). The model for cluster 1 included the percent urban land use in a 0.5 km buffer which was negatively correlated with BU disease prevalence, a result similar to the country-wide analysis. Examination of the residuals from the model of cluster 1 did not indicate autocorrelation among residuals (Figure 2B).

## Discussion

### Association of Buruli ulcer disease prevalence and land use/cover

Our top-ranked model indicated that low elevation, rural villages surrounded by forest land cover, and with drainage basins demonstrating variable wetness patterns were most likely to have high BU disease prevalence rates. These results supported our predictions, except for the positive relationship between forest land cover and BU disease prevalence. The positive relationship between forest land cover and BU disease prevalence was unexpected, largely because most reviews of BU disease do not associate forest cover with higher prevalence [12,43]. Our analysis suggests that variation in forested land cover is an important factor for BU disease, but the reasons for this are not clear. However, three explanations may be that (1) remnant forests offer habitat for the yet unidentified vectors and/or reservoirs of *M. ulcerans*, (2) that the forests provide conditions or resources useful to pathogen proliferation in routinely used water sources, and (3) the resolution of our land use/cover data resulted in agricultural areas to be classified as 'forest' (e.g., teak or cocoa plantations; see Study limitations and future directions below). The first potential explanation is plausible. For example, a study from Australia suggests that marsupials may be acting as hosts and mosquitoes as mechanical vectors of BU disease [44]. Such a terrestrial-aquatic link would suggest that suitable terrestrial conditions, such as forest cover, could play a role in BU outbreaks. This is an area of research that will need to be studied in greater detail as the vectors and reservoirs are identified. The second explanation is also possible, since *M. ulcerans *has been found in a variety of aquatic taxa and habitats [45-47]. Most recently, a study in Ghana has identified *M. ulcerans *in water filtrate, on the biofilm of substrates and associated with aquatic invertebrates in water bodies associated with human domestic uses [47]. However, how the surrounding terrestrial environment might mediate *M. ulcerans *remains unknown. In addition, to date there are no field studies that provide information on the important environmental growth conditions of *M. ulcerans *in aquatic habitats, making inferences about terrestrial-aquatic habitat linkages difficult.

Our findings also suggest that villages with higher BU disease prevalence rates are located at low elevation areas with variable wetness patterns. The negative relationship between BU prevalence and elevation may be related to potentially high-risk human activities that occur at lower elevations, such as farming. Duker et al. [23] noted that most farmland in Amansie West district, Ghana, is located at low elevation, such as floodplains, because they are often fertile and easy to irrigate. Farming near aquatic ecosystems, especially while not wearing protective clothing, has been shown to be a risk factor for BU [16,48]. Villages in low elevation areas may be prone to large changes in moisture conditions due to flooding. In fact, flooding has been associated with BU disease outbreaks in Africa and Australia [12,43,44]. We propose that the surrounding land cover and topography dictate the extent and duration of village flooding, thereby affecting human exposure potential to the pathogen, and perhaps influencing conditions that are necessary for *M. ulcerans *proliferation as discussed by Merritt et al. [12].

Urban land cover was negatively correlated with BU disease prevalence in the country-wide analysis. This result supports earlier work, where the percent urban land cover in a 50 km buffer around individual villages was negatively correlated to the probability of BU disease presence in Benin [34]. In addition, the proportion of urban land use in 0.5 km buffer was also negatively correlated with BU disease prevalence for the primary cluster (cluster 1) in the disease cluster analysis. We speculate that this negative correlation between urban land use and BU disease prevalence is due to increased availability of pumped water (i.e., protected water sources), better sanitation in urban environments or the elimination of unknown habitat or reservoirs of the pathogen. This supports findings from case-control studies [15]; however, the availability of pumped water is likely not the only factor since some populations do not use pumped water and prefer using non-protected water sources. An additional hypothesis is that small urban centres provide additional employment opportunities that replace the need for subsistence or other types of farming, an increasing trend in Benin [49,50]. These additional employment opportunities would lead to lower contact rates with higher risk habitats compared to rural areas, because agricultural activities in rural areas increase the probability and intensity of exposure to *M. ulcerans*.

**Figure 4:**
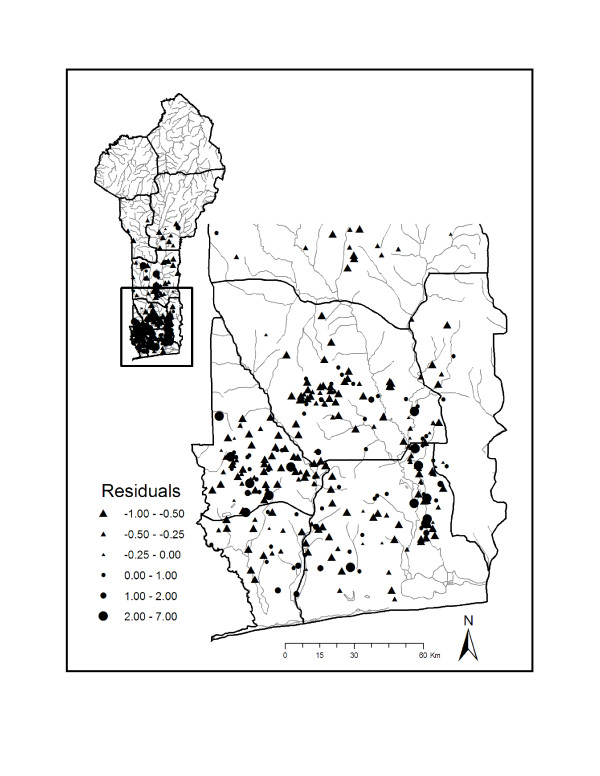
**Residuals from negative binomial regression model**. Residuals (residuals = [observed – fitted]/sd, where $\text{sd}=\sqrt{fitted\times (1+fitted)/\alpha }$ where *α *is the estimated negative binomial parameter) for each village from the top-ranked model. Negative residuals are represented by triangles and positive residuals are represented by circles.

In addition to identifying correlates of BU disease prevalence, our model also allows for the identification of villages that have greater or lower than expected prevalence rates compared to predicted values. Examining a plot of residuals from the model allows for the identification of villages whose prevalence rates were either overestimated or underestimated by the model (Figure 4). Villages with relatively large positive or negative residuals represent villages that may prove useful for elucidating important drivers of BU disease. For example, studying social, economic, and ecological factors of villages predicted to have low BU prevalence rates, but have high observed BU prevalence rates in comparison to villages which show the opposite pattern may lend further insight into the ecology and transmission of BU disease.

### Disease Clusters

We identified five significant BU disease clusters, one primary cluster with greater than expected prevalence rates, and four secondary clusters: two with greater than expected prevalence and two with lower than expected prevalence rates. The disease clusters with greater than expected prevalence rates span from the western to the eastern borders of southern Benin. The villages with lower than expected prevalence include villages near the coast and in the south-central area of Benin. Although, we did not find a significant relationship between the distance to the nearest river or percent water in our analyses (see Study limitations and future directions), if the transmission of BU disease is associated with aquatic habitats, the pattern of these clusters may be partly explained by the location of the villages to aquatic habitats. The clusters with greater than expected prevalence include villages in relatively close proximity to the Zou, Ouémé, and Couffo rivers. The clusters include endemic districts including Ze in Atlantic, Ouinhi in Zou, and Lalo in Couffo. In addition, this geographic region is dominated by agriculture including maize, cassava, and beans. The presence of aquatic habitats and agricultural activities supports previous hypotheses with regards to risk factors for BU disease (e.g., [48]). The cluster with lower than expected prevalence rates in the Collines district (cluster 5) may be driven by the semi-arid conditions found as you move north in Benin. The villages near the coast, with lower than expected prevalence, may be due to increased urbanization near the coastal areas, and thus related to our hypothesis regarding access to pumped water sources in urban settings.

Although our analyses focused on map-based landscape data, it is likely that human behaviour is also an important element in the transmission of BU disease. Several case-control studies have documented that human behaviour is an important risk factor for transmission, but results have not been consistent [17,18,48]. In addition, the habitat and reservoirs for *M. ulcerans *are just now being studied, with improved molecular diagnostic tools for environmental samples being developed and refined. A better understanding of reservoir-host dynamics is essential for understanding and modelling the dynamics of BU disease, with research expanding to examine aquatic-terrestrial linkages and the importance of recent land use modifications associated with focal outbreaks. The results presented here identified specific geographic areas where future research can direct efforts into understanding host-vector dynamics.

### Study limitations and future directions

Land use/cover covariates that were important in predicting BU disease prevalence included percent urban (negatively correlated) and forest (positively correlated) measured in a 20 km buffer width around each village. These results do not suggest that smaller-scale patterns of land use/cover are not important, rather they likely reflect a limitation of our remotely sensed data, namely the relatively coarse resolution of the Landsat ETM+ data (30 m^2^). For example, we were unable to quantify land use heterogeneity at smaller scales, resulting in relatively little variation in our covariates at small buffer widths. It should also be noted that an accuracy assessment of the LULC data used in this study was not conducted because ground-truth data were not available, primarily due to the cost and time involved in quantifying LULC classification error for the entire southern portion of Benin. However, we did take steps to minimize the potential for classification errors to influence our results. For example, we used only easily distinguished LULC classes in our study (e.g., forest, urban, and water), which have fewer propensities for errors during the classification process [51]. In addition, we did not consider LULC categories that were comprised of more than two LULC types in an effort to reduce the amount of classification error potentially introduced into our analyses. Finally, the ecology of BU disease is hypothesized to be linked to aquatic environments. Although our study did not find significant relationships between a village's distance to the nearest river or percent water in surrounding buffers, this should not be interpreted as reducing the likelihood of a link between BU disease and aquatic environments. Again, the resolution of our data may have resulted in the inability to quantify small bodies of water that may be important water sources for villages. In addition, we did find a positive relationship between BU prevalence and the standard deviation of the wetness index, suggesting that wetness patterns, and thus aquatic habitats, warrant additional research. From this study, and others like it, we can expand to field-based studies that include finer resolution land use/cover data with an accuracy assessment. The use of higher resolution satellite imagery with ground truthing and aerial photos are warranted to examine land use/cover and Buruli ulcer disease prevalence at small spatial scales, and our disease cluster analyses identified specific geographic locations for such targeted investigations.

## Conclusion

The mode of transmission for BU disease remains unknown. However, large-scale environmental studies such as this one are important for quantifying broad scale patterns of disease emergence with associated landscape variables. The results of such studies assist in identifying specific geographic areas for more focused research efforts, thereby providing a scientifically-based and data-driven approach to understanding the ecology of this neglected disease. Our study accomplishes this by identifying complex interactions of land use/cover and topography that are important for understanding broad patterns of disease emergence at the country scale. Our results also suggest that aquatic habitat-landscape interactions should be investigated when attempting to identify high risk environments and potential hosts and vectors. This study also identifies disease clusters, indicating specific geographic areas for more focused research efforts. A combination of large-scale environmental assessments with complementary local-scale epidemiological studies is a recommended approach to understanding the emergence of understudied and neglected diseases such as BU disease.

## Competing interests

The authors declare that they have no competing interests.

## Authors' contributions

TW and TOB performed statistical analyses and participated in writing the manuscript, MEB participated in writing of the manuscript, interpretation, and providing funding, JQ was responsible for the remote sensing data acquisition and analysis, and RCJ provided BU disease case data and geographic coordinates for all villages in Benin.

## Supplementary Material

Additional file 1 AICc weights and strength of evidence for negative binomial regression candidate models. The file provides AICc weights and strength of evidence for negative binomial regression candidate models used to investigate the relationships between land use/cover covariates and Buruli ulcer disease prevalence rates. Click here for file
