# Supplementary material for: Buruli ulcer disease prevalence in Benin, West Africa: associations with land use/cover and the identification of disease clusters
Source: Int J Health Geogr. 2008 May 27;7:25. doi: 10.1186/1476-072X-7-25 (PMC2423183; doi:10.1186/1476-072X-7-25)
Supplement: Additional file 1 — AICc weights and strength of evidence for negative binomial regression candidate models. The file provides AICc weights and strength of evidence for negative binomial regression candidate models used to investigate the relationships between land use/cover covariates and Buruli ulcer disease prevalence rates. [file 1476-072X-7-25-S1.doc]

**AICc weights and strength of evidence for negative binomial regression candidate models.** Response variable is the number of Buruli ulcer cases in villages in Benin, West Africa (*n* = 327 villages). All models contained $\log(N_{ij})$as a covariate, where = $N_{ij}$is the total population for village *i* in district *j*, as an offset term and a random effect for the district where each village was located (see equation 1). The null model is a model that includes only an intercept and the offset term. Models are sorted descending based on AICc weights. ag = agricultural land use, elevation = mean village elevation. All land use/cover types are proportions within a 20 km buffer of each village. See Table 1 for summary of covariates.

| Model | AICc | AICc weight | Strength of evidence |
| --- | --- | --- | --- |
| urban+forest+elevation+standard deviation of the wetness index | 1114.6 | 1.00 | 1.00 |
| urban+elevation+standard deviation of the wetness index | 1116.2 | 2.3 | 2.28 |
| urban+elevation | 1117.4 | 5.4 | 5.42 |
| urban+forest+elevation | 1118.3 | 6.5 | 6.50 |
| urban+ag+elevation | 1120.0 | 15.3 | 15.32 |
| urban+forest+elevation+wetland | 1120.3 | 17.7 | 17.71 |
| urban+forest+elevation+wetland+distance to river | 1121.2 | 28.1 | 28.06 |
| elevation | 1124.4 | 137.7 | 1.38E+02 |
| urban+forest+ standard deviation of the wetness index | 1126.5 | 400.3 | 4.00E+02 |
| urban+ standard deviation of the wetness index | 1129.4 | 1.7E+03 | 1.71E+03 |
| urban | 1131.4 | 4.6E+03 | 4.56E+03 |
| urban+ag | 1131.5 | 4.7E+03 | 4.68E+03 |
| distance to river+wetness index | 1139.0 | 2.0E+05 | 2.03E+05 |
| forest | 1139.1 | 2.1E+05 | 2.10E+05 |
| ag | 1140.6 | 4.5E+05 | 4.48E+05 |
| distance to river | 1141.4 | 6.8E+05 | 6.75E+05 |
| standard deviation of the wetness index | 1141.7 | 7.9E+05 | 7.93E+05 |
| wetland | 1141.8 | 8.3E+05 | 8.34E+05 |
| wetness index | 1141.9 | 8.9E+05 | 8.88E+05 |
| random intercept | 1145.4 | 5.1E+06 | 5.10E+06 |
